# Identification of Four Immune Subtypes in Bladder Cancer Based on Immune Gene Sets

**DOI:** 10.3389/fonc.2020.544610

**Published:** 2020-10-05

**Authors:** Chaozhi Tang, Jiakang Ma, Xiuli Liu, Zhengchun Liu

**Affiliations:** ^1^Department of Urology, The First Affiliated Hospital of China Medical University, Shenyang, China; ^2^Department of Oncology, The Second Affiliated Hospital of Zhengzhou University, Zhengzhou, China; ^3^Department of Oncology, Affiliated Hospital of Guilin Medical University, Guilin, China; ^4^Department of Radiation Oncology, Affiliated Hospital of Guilin Medical University, Guilin, China

**Keywords:** immunotype, bladder cancer, immune cell, gene set enrichment analysis, prognosis

## Abstract

Molecular classification of bladder cancer is becoming increasingly important for its clinical management. And, the current classifications are primarily based on gene expression profiles. We identified four immunotypes of bladder cancer (referred to as C1–C4) based on gene expression profiles performed by immune-related gene sets in three independent data sets, and proved that this classification is effective and reproducible. We found that C2 is an immune-infiltrating type and C4 is an immune “desert” type. These types are characterized by the up- and downregulation of genes encoding numerous immune checkpoint proteins and HLA and regulating human immune cell subgroups. The survival rate was better for the C2 subtype than for other subtypes. We believe that this can be explained by the antitumor effects of CD4 memory T cells and CD8 T cells as well as their ability to circumvent M0 macrophage antitumor immunity. In addition, C2 was most sensitive to not only anti-PD-1 immunosuppressive therapy, but also conventional chemotherapeutics such as gemcitabine and bleomycin. The C4 subtype was most sensitive to the chemotherapy drugs cisplatin and doxorubicin. This theoretical framework may guide the personalized treatment of bladder cancer in the future. It is worth noting that the C2 immune infiltration type positively correlates with a variety of stromal components, such as enrichment of endothelial cells and fibroblasts, epithelial-mesenchymal transition, and angiogenesis, together with enrichment of seven kinds of stem cells. We further identified tumor-related JAK-STAT and other signaling pathways in the C2 subtype, along with important mutations in the proteins involved in these pathways, revealing the complex mechanism underlying tumor immune escape. Our results, and particularly the identification of hub genes specific to the C2 and C4 subtypes, provide a reference for the development of immunotherapeutic agents against bladder cancer.

## Introduction

Bladder cancer (BLCA) is a complex disease with high morbidity and mortality, with at least 430,000 cases globally diagnosed each year (1–3). Despite considerable progress in the treatment of BLCA, such as the development of transurethral cystectomy and intravesical Bacillus Calmette-Guérin chemotherapy for non-muscle-invasive BLCA, and radical cystectomy for muscle-invasive BLCA, two-thirds of patients with invasive urothelial BLCA show relapse or disease progression within 5 years (4). The poor prognosis and recurrence of BLCA are largely due to its heterogeneity. The molecular and genetic characteristics of tumor cells determine the aggressiveness and sensitivity to treatment (5). Therefore, the integration of molecular subtypes into the clinical management of BLCA is critical (6). Robertson et al. (7) identified five BLCA subtypes based on RNA-seq data, and noted that the basal squamous subtype has the highest levels of T cell markers, inflammatory genes, and lymphocyte infiltration. Patients with luminal subtypes benefit the most from anti-PDL1 therapy, with increased expression of multiple immune markers, including *CD274* (*PD-L1*) and *PDCD1* (*PD-1*). Despite many studies on the molecular characteristics of BLCA based on gene expression profiles, methylation patterns, and mutation distributions, few studies have directly investigated the immunotypes of BLCA.

Therapies that modulate the immune response offer substantial benefits against many cancer types (8–13). Several immune checkpoint inhibitors targeting programmed cell death protein 1 (*PD1*) and its ligands *PDL1* and cytotoxic T lymphocyte-associated protein 4 (*CTLA4*) have been approved for BLCA. These advances provide opportunities for precision and personalized treatment. However, *PD1*/*PD-L1* therapy, currently the most well-established approach, is only beneficial in ~20% of patients. This may be explained by the abnormally activated Treg cells. In cancer, Treg cells secrete a variety of inhibitory cytokines after T cell receptor (TCR)-mediated signaling activation, allowing tumor cells to escape immune surveillance (14). This mechanism of tumor immune escape highlights the challenges in effective implementation of immunotherapy.

In this study, we identified four distinct and stable subtypes from The Cancer Genome Atlas (TCGA) BLCA cohort based on immune gene sets and verified these subtypes with additional cross-platform databases, GEO, and ArrayExpress. The distribution of the four subtypes with respect to clinical traits, prognostic significance, and the pathways related to subtype-specific heterogeneity were evaluated. Finally, we investigated the associations of the four subtypes with human immune cell populations and characteristic genes. These results provide a powerful basis for future BLCA immunotherapy.

## Methods

### Ethics Statement

All data were downloaded from public databases and, therefore, did not require approval and review by the ethics committee.

### Data Processing

The BLCA datasets were obtained from three platforms, TCGA, GEO, and ArrayExpress. RNA-seq data (FPKM), variant data of varscan, and clinical information for 407 cases were downloaded from TCGA Knowledge Base (https://portal.gdc.cancer.gov/repository), UCSC Xena (https://xenabrowser.net/datapages/), and cBioPortal for Cancer Genomics (http://www.cbioportal.org/). Gene annotation was performed using the Ensemble database. RNA-seq (i.e., FPKM values) and clinical data (*N* = 476) from the European Genome-phenome Archive (EGA) for 476 cases of early urothelial carcinoma (E-MTAB-4321) were downloaded from the ArrayExpress (https://www.ebi.ac.uk/arrayexpress/) database. The GEO BLCA dataset was merged from the following four datasets: GSE13507 (165 cases of primary BLCA), GSE32548 (*N* = 131), GSE31684 (*N* = 93), and GSE48276 (*N* = 116). The genes were annotated using the data files for Illumina Human-6 v2.0 Expression BeadChip, Illumina HumanHT-12 V3.0 Expression BeadChip, [HG-U133_Plus_2] Affymetrix Human Genome U133 Plus 2.0 Array, and Illumina HumanHT-12 WG-DASL V4.0 R2 Expression BeadChip. The expression data for the four datasets were all quantile normalized, and batch effect correction was performed using the sva package in R.

### Identification of BLCA Subtypes Based on Immune Gene Sets

A literature search was performed to determine 29 immune gene sets (15) to represent tumor immunity. For each BLCA dataset, the GSVA package was used for ssGSEA of the 29 immune gene sets. The ConsensusClusterPlus package was used for consensus clustering and molecular subtype screening of ssGSEA scores. In brief, k-means clustering was used, with 50 iterations (each using 80% of the samples). The best cluster number was determined by the clustering score for the cumulative distribution function (CDF) curve, and the relative changes in the area under the CDF curve were evaluated. Principal component analysis (PCA), which is often used for dimensionality reduction, was used to verify the reliability of the consensus clusters.

### Heatmap

The ssGSEA score *x*_i_ for each BLCA sample i was converted to ${x_{\text{i}}}^{\prime }$ using the equation ${x_{\text{i}}}^{\prime }$ = (*x*_i_-*x*_min_)/(*x*_max_-*x*_min_), where *x*_max_ and *x*_min_ represent the maximum and minimum ssGSEA scores for all samples in the BLCA dataset, respectively. The pheatmap package in R was used for heatmap visualization.

### Evaluation of Immune Cell Infiltration, Tumor Purity, and Matrix Content in BLCA

The ESTIMATE algorithm (16) uses transcriptome data to infer the tumor cell composition and infiltration and to identify specific signatures related to the infiltration of stromal cells and immune cells. The algorithm was implemented using the estimate package in R. Differences in molecular subtypes of BLCA in each dataset were compared using the Kruskal–Wallis test.

### Survival Analysis

Kaplan–Meier curves were used to evaluate survival time in patients with BLCA in each dataset. The survival probability, including overall survival (OS), relapse-free survival (RFS), and progression-free survival (PFS), were evaluated for patients with BLCA. The log-rank test was used with *P* < 0.05 as the threshold for significance.

### Comparison of Immune Cell Subgroups Among Molecular Subtypes of BLCA

CIBERSORT (17) is a tool for the deconvolution of the expression matrix of immune cell subgroups based on the principle of linear support vector regression. The CIBERSORT package in R was used to evaluate differences in the frequencies of 22 immune cell types among BLCA molecular subtypes.

### Gene Co-expression Network Analysis

To investigate the distribution of characteristic immune genes in each molecular subtype of BLCA and to identify genes or gene modules that are highly related to immune cell infiltration, the WGCNA R package was used to evaluate data from IMMPORT (https://www.immport.org/) for 1,671 immune-related genes consisting of the expression matrix. WGCNA network construction and module detection use an unsigned topological overlap matrix. The optimal soft threshold (power) was 4, the minimum number of genes in the module was 30, and the branch merge interception height was 0.25. A hub gene was defined as a gene with a connection weight > 0.30 and no < 10 connected genes. The gene co-expression network was visualized using Cytoscape 3.7.1. The R package “survminer” was used to visualize the survival curve based on the best cut-off value for the respective gene.

### Gene Set Enrichment Analysis (GSEA)

The expression matrices for samples classified as subtypes C2 and C4 in all three datasets were used for a GSEA using c2.cp.kegg.v7.0.symbols.gmt as the reference gene set. Using GSEA version 4.0, the number of permutations was set to 1,000, and FDR < 0.05 was the screening threshold.

### Immune and Chemical Response Prediction

We used TCGA's FPKM RNA seq expression profile combination subclass mapping method to predict the clinical response of BLCA immune subtypes to immune checkpoint blockade. Based on the largest publicly available pharmacogenomics database [Genomics of Drug Sensitivity in Cancer (GDSC), https://www.cancerrxgene.org/], we predicted the chemotherapy response of each sample. Four commonly used drugs were selected: cisplatin, doxorubicin, gemcitabine, and bleomycin. The prediction process was carried out using the R package “pRRophetic,” where the half-maximum inhibitory concentration IC50 of the sample was estimated using ridge regression, and the accuracy of the prediction was evaluated using 10-fold cross-validation, according to the GDSC training set. All parameters were set as the default values, and the repeated gene expression was averaged.

### Statistical Analysis

The association between routine clinical variables and immune subtypes was tested using the chi-square test or Fisher's exact test, and Benjamini & Hochberg's FDR corrected multiple tests. Kaplan–Meier curve and log-rank test were used to compare OS, RFS, and PFS of different immune subtypes. Mann–Whitney *U-*test was used to compare the expression of immune checkpoint proteins between different subtypes, and the difference in IC50 and stromal and stem cells among the different subtypes was tested using the Kruskal–Wallis test. All statistical tests were bilateral-sided and implemented in R programing language.

## Results

### Identification of BLCA Subtypes Based on Immune Gene Sets

**Figure 1:**
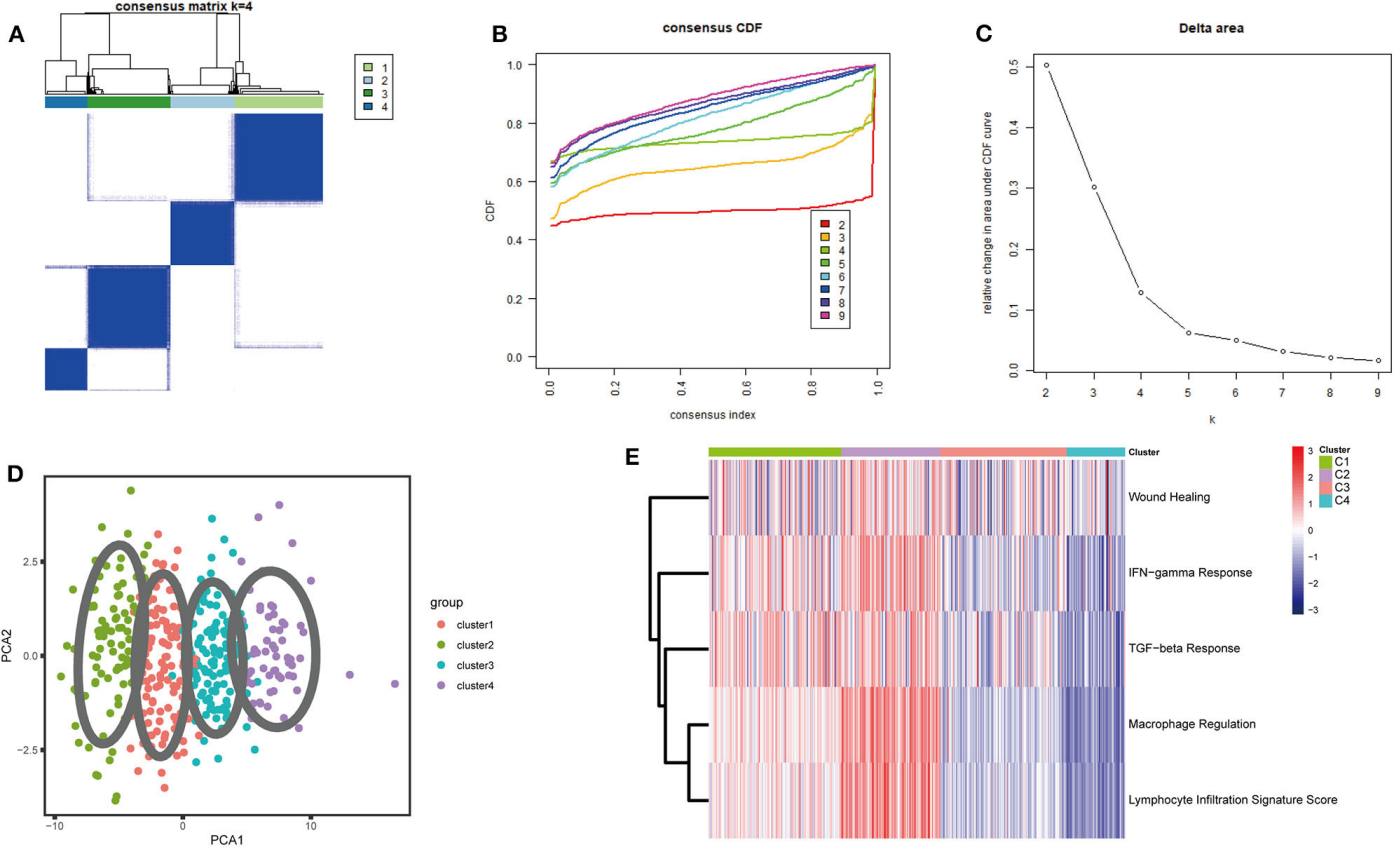
Consensus clustering of BLCA TCGA cohorts. **(A)** The consensus score matrix for BLCA samples when *k* = 4. A higher consensus score between two samples indicates that they are more likely to be assigned to the same cluster in different iterations. **(B)** Cumulative distribution function (CDF) curve of the consistency score for different subtype numbers (*k* = 2–9). **(C)** Delta area plot of the relative increase in cluster stability when *k* = 4. **(D)** Principal component analysis (PCA) of all samples based on ssGSEA scores; each point represents a sample, and different colors distinguish the subtypes. **(E)** Heatmap of five immune characteristics for four subtypes (C1–C4).

A set of 29 immune-related genes representing multiple immune cell types, functions, and pathways was used to study BLCA subtypes in TCGA. A single-sample GSEA (ssGSEA) was performed to obtain scores for 29 immune gene sets in each sample. The R package ConsensusClusterPlus was used to divide all tumor samples into k subtypes (*k* = 2–9). Based on the consensus score of the CDF curve, *k* = 4 was optimal. In addition, PCA showed that the ssGSEA scores based on the 29 immune gene sets could be divided into four subtypes (referred to as C1–C4; Figures 1A–D). The extent of immune infiltration decreased in the following order: C2 > C1 > C3 > C4 (Supplementary Figure 1). Consistent results were obtained using the GEO and ArrayExpress validation sets.

### Immune Characteristics of the Four Immune Subtypes

**Figure 2:**
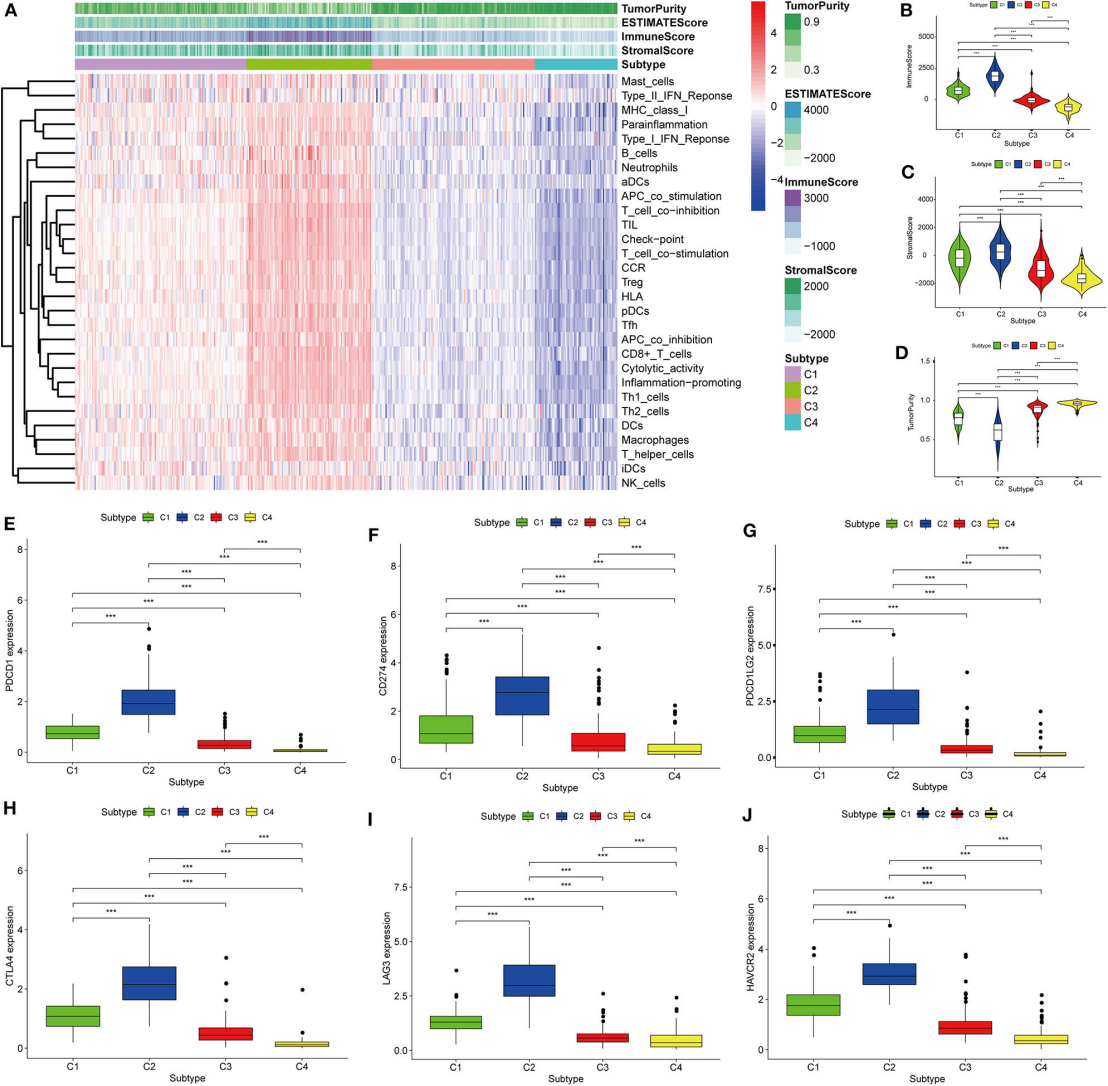
Identification of the four immune subtypes in the BLCA TCGA cohort. **(A)** Heatmap of the four immune subtypes based on ssGSEA scores for 29 immune gene sets. **(B–D)** Evaluation of stromal scores, immune scores, and tumor purity for the four immune subtypes by Mann–Whitney *U-*test. **(E–J)** Differential expression of the immune checkpoint genes *PDCD1, CD274, PDCD1LG2, CTLA4, LAG3*, and *HAVCR2* among the four subtypes evaluated by Mann–Whitney *U-*test; bars indicate medians. ***P < 0.001.

**Figure 3:**
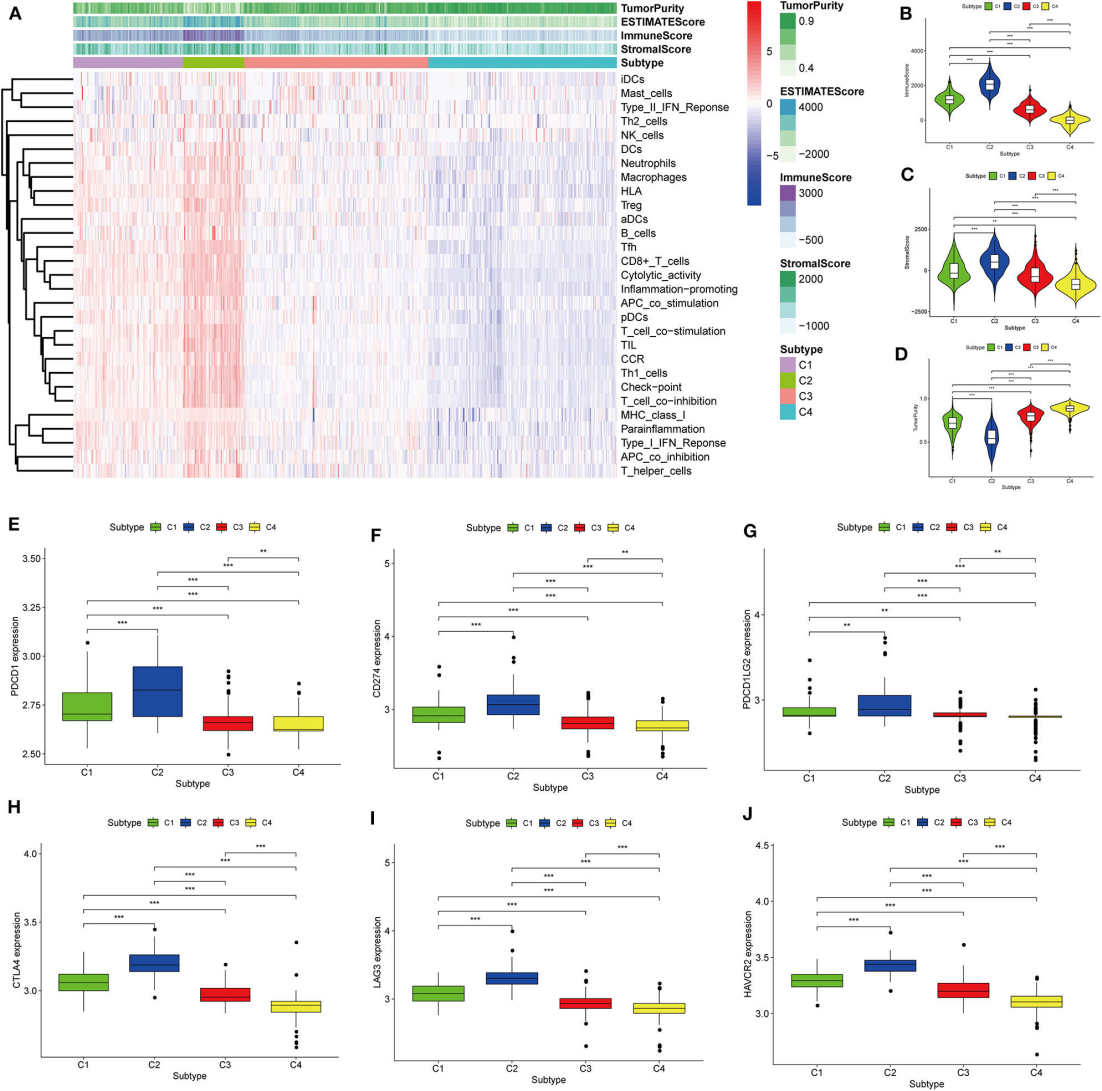
Identification of the four immune subtypes in the BLCA GEO cohort. **(A)** Heatmap of the four immune subtypes based on ssGSEA scores for 29 immune gene sets. **(B–D)** Evaluation of stromal scores, immune scores, and tumor purity for the four immune subtypes by Mann–Whitney *U-*test. **(E–J)** Differential expression of the immune checkpoint genes *PDCD1, CD274, PDCD1LG2, CTLA4, LAG3*, and *HAVCR2* among the four subtypes, as evaluated by Mann–Whitney *U-*test; bars indicate medians. **P < 0.01, ***P < 0.001.

**Figure 4:**
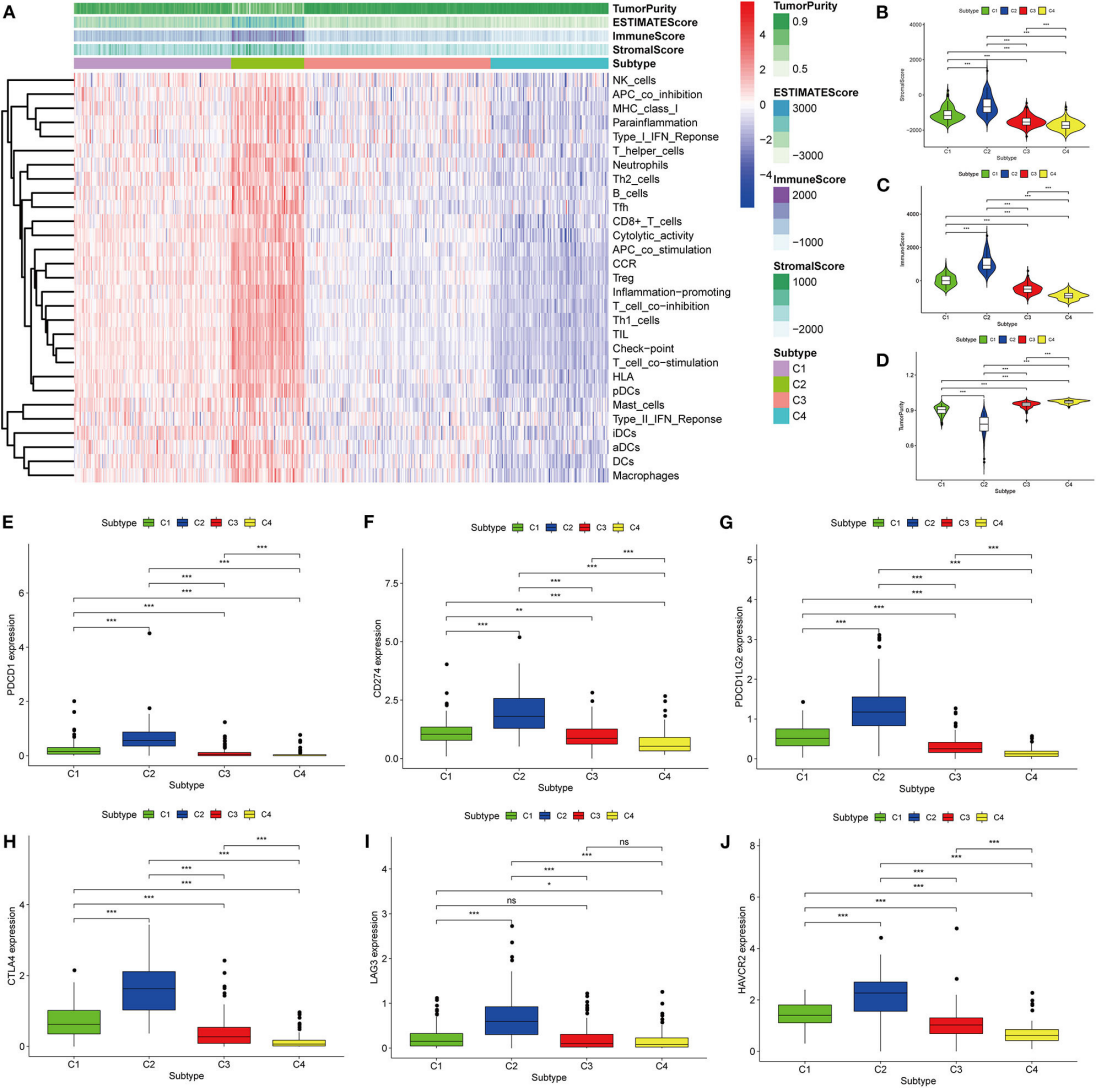
Identification of the four immune subtypes in the BLCA ArrayExpress cohort. **(A)** Heatmap of the four immune subtypes based on ssGSEA scores for 29 immune gene sets. **(B–D)** Evaluation of stromal scores, immune scores, and tumor purity for the four immune subtypes by Mann–Whitney *U-*test. **(E–J)** Differential expression of the immune checkpoint genes *PDCD1, CD274, PDCD1LG2, CTLA4, LAG3*, and *HAVCR2* among the four subtypes, as determined by Mann–Whitney *U-*test; bars indicate medians. *P < 0.05, **P < 0.01, ***P < 0.001, ns: no significance.

Thorsson et al. (18) defined 33 types of non-hematological tumors in TCGA based on the immune expression characteristics of five core modules (wound healing, IFN-gamma response, TGF-beta response, macrophage regulation, and lymphocyte infiltration) including 160 immune characteristics. Using three cross-platform data sets, we obtained four stable immune subtypes. C2 had the highest degree of infiltration based on the five core modules, followed by C1, C3, and C4. In particular, C4 exhibited a “desert” -like phenotype (Figure 1E) that lacked T cells, especially CD8 T cells, in the tumor microenvironment. This is consistent with the results of our ssGSEA clustering analysis based on 29 immune gene sets, indicating that the degree of infiltration for most immune sets decreases in the following order: C2 > C1 > C3 > C4 (Figures 2A, 3A, 4A). For example, C2 had the highest ssGSEA scores for gene sets related to B cells, immune checkpoints, HLA (Supplementary Figure 2), CD8+ T cells, cytolytic activity, Th1 cells, macrophages, and other immune components, and C4 had the lowest ssGSEA scores. ESTIMATE was used to assess the stromal score, immune score, and tumor purity of the four subtypes. For all three cross-platform datasets, C2 had the highest stromal and immune scores, and C4 had the lowest stromal and immune scores (C2 > C1 > C3 > C4). In contrast, C2 had the lowest tumor purity, while C4 had the highest tumor purity (C4 > C3 > C1 > C2) (Figures 2B–D, 3B–D, 4B–D). Finally, we examined the expression of six immune checkpoint genes (i.e., *PDCD1, CD274, PDCD1LG2, CTLA4, HAVCR2*, and *LAG3*), which are related to immune escape. With respect to immunotypes, the expression levels of genes decreased in all data sets in the following order: C2 > C1 > C3 > C4 (Figures 2E–J, 3E–J, 4E–J).

### Clinical Characteristics of the Four Immune Subtypes

To explore the association between immunotyping and common clinical features, we analyzed sex, age, grade, pathological subtype, T stage, M stage, N stage, race, lymphatic invasion, and metastasis in the TCGA cohort (Table 1). Tumor grade differed significantly among the four immunotypes. In particular, the proportion of low-grade tumors was significantly higher in C4 cases than that in the other subtypes, and the proportion of high-grade tumors was significantly higher in C1 and C2 than in the other subtypes (FDR = 0.0030). Similarly, with respect to the pathological subtype of BLCA, non-papillary lesions were more frequent in C1 and C2, and papillary lesions were more frequent in C4 (FDR < 0.0001). Further analysis of the TNM system revealed that the proportion of M0 cases was higher in C4 than in other subtypes, and the proportion of M1 cases was higher in C3 than in other subtypes (FDR = 0.0127). Regarding race, C4 was disproportionately found in the Asian population, C1 was most frequent in the population of African descent, and C2 was most frequent in the population of European descent (FDR < 0.0001). The percentage of lymphovascular invasion-negative cases was highest for C2, and the proportion of lymphovascular invasion-positive cases was highest for C1 among all subtypes. There were no significant differences in the distribution of immunotypes with respect to other clinical characteristics. In the ArrayExpress validation cohort (Table 2), only the grade and pathological subtypes showed significant differences among immunotypes. Low-grade tumors were more common in C1, and high-grade tumors were more common in C2 than in other subtypes (*P* = 0.0174). Papillary lesions were more common in C4, and non-papillary lesions were more common in C2 than in other subtypes, and C1 had the highest mixed component (*P* = 0.0213). No other clinical traits differed significantly among immunotypes.

**Table 1:** Correlations between the four immune subtypes and clinical characteristics in the TCGA cohort.

**Parameter**		**Immune subtypes (n, %)**				***P*-value**	**FDR**
		**C1**	**C2**	**C3**	**C4**		
Sex	Male	95 (72.5)	64 (67.4)	92 (76.0)	49 (81.7)	0.2247	0.2475
	Female	36 (27.5)	31 (32.4)	29 (24.0)	11 (18.3)		
Age	≤65 years	53 (40.5)	33 (34.7)	56 (46.3)	18 (30)	0.13577	0.1659
	>65 years	78 (59.5)	62 (65.3)	65 (53.7)	42 (70)		
Grade	Low grade	2 (1.5)	1 (1.0)	9 (7.4)	9 (15)	**0.0008**	**0.0030**
	High grade	129 (98.5)	93 (98.0)	110 (90.9)	51 (85)		
	NA	0 (0)	1 (1.0)	2 (1.7)	0 (0)		
Stage	Stage I	0 (0)	0 (0)	1 (0.8)	1 (1.7)	0.1119	0.1539
	Stage II	36 (27.5)	33 (34.7)	34 (28.1)	26 (43.3)		
	Stage III	42 (32.1)	38 (40.0)	41 (33.9)	19 (31.7)		
	Stage IV	53 (40.4)	24 (25.3)	44 (36.4)	13 (21.6)		
	NA	0 (0)	0 (0)	1 (0.8)	1 (1.7)		
Subtype	Non-papillary	101 (77.1)	75 (78.9)	67 (55.4)	27 (45.0)	**<0.0001**	**<0.0001**
	Papillary	28 (21.4)	19 (20.0)	52 (42.9)	33 (55.0)		
	NA	2 (1.5)	1 (1.1)	2 (1.7)	0 (0)		
T	T0	0 (0)	0 (0)	0 (0)	1 (1.7)	0.0695	0.1207
	T1–2	33 (25.2)	30 (31.6)	38 (31.4)	20 (33.3)		
	T3–4	91 (69.5)	60 (63.2)	72 (59.5)	30 (50)		
	NA	7 (5.3)	5 (5.2)	11 (9.1)	9 (15)		
M	M0	60 (45.8)	34 (35.8)	66 (54.5)	35 (58.3)	**0.0046**	**0.0127**
	M1	3 (2.3)	0 (0)	6 (5.0)	2 (3.3)		
	Mx	67 (51.1)	61 (64.2)	49 (40.5)	22 (36.7)		
	NA	1 (0.8)	0 (0)	0 (0)	1 (1.7)		
N	N0	69 (52.7)	61 (64.2)	64 (52.9)	42 (70)	0.0768	0.1207
	N1–3	52 (39.7)	24 (25.3)	41 (33.9)	12 (20)		
	Nx	8 (6.1)	10 (10.5)	14 (11.5)	4 (6.7)		
	NA	2 (1.5)	0 (0)	2 (1.7)	2 (3.3)		
Race	Asian	9 (6.9)	4 (4.2)	14 (11.6)	16 (26.7)	**<0.0001**	**0.0001**
	Black	9 (6.9)	6 (6.3)	4 (3.3)	4 (6.7)		
	White	110 (83.9)	85 (89.5)	94 (77.7)	35 (58.3)		
	NA	3 (2.3)	0 (0)	9 (7.4)	5 (8.3)		
Lymphovascular invasion	No	29 (22.1)	43 (45.3)	35 (28.9)	23 (38.3)	**0.0100**	**0.0219**
	Yes	59 (45.0)	27 (28.4)	47 (38.8)	17 (28.3)		
	NA	43 (32.9)	25 (26.3)	39 (32.3)	20 (33.4)		
Metastasis	Metastatic	30 (22.9)	11 (11.6)	21 (17.4)	8 (13.3)	0.3692	0.3692

*T, tumor; M, metastasis; N, lymph node. P-values were obtained by Fisher's exact test; FDR was corrected by the Benjamini & Hochberg methods*.

*Bold values indicate statistical significance in both P-value and FDR, that is, < 0.05*.

**Table 2:** Correlation between the four immune subtypes and clinical characteristics in the ArrayExpress cohort.

**Parameter**		**Immune subtype (n, %)**				***P*-value**	**FDR**
		**C1**	**C2**	**C3**	**C4**		
Sex	Male	106 (75.7)	47 (72.3)	129 (77.7)	85 (81.0)	0.5901	0.6725
	Female	34 (24.3)	18 (27.7)	37 (22.3)	20 (19.0)		
Age	≤65 years	53 (37.9)	19 (29.2)	62 (37.3)	35 (33.3)	0.5895	0.6725
	>65 years	87 (62.1)	46 (70.8)	104 (62.7)	70 (66.7)		
Grade	High grade	46 (32.9)	30 (46.2)	74 (44.6)	42 (40.0)	0.0174	0.0960
	Low grade	88 (62.8)	35 (53.8)	92 (55.4)	62 (59.0)		
	PUNLMP	6 (4.3)	0 (0)	0 (0)	1 (1.0)		
T	CIS	1 (0.7)	1 (1.5)	1 (0.6)	0 (0)	0.1642	0.3694
	Ta	112 (80)	44 (67.7)	111 (66.8)	78 (74.3)		
	T1	24 (17.2)	15 (23.1)	49 (29.5)	24 (22.8)		
	T2–4	3 (2.1)	5 (7.7)	5 (30.1)	3 (2.9)		
Subtype	Papillary	119 (85.0)	51 (78.5)	146 (88.0)	101 (96.2)	0.0213	0.0960
	Solid	2 (1.4)	3 (4.6)	6 (3.6)	3 (2.9)		
	Mixed	4 (2.9)	1 (1.5)	3 (1.8)	0 (0)		
	Unknown	15 (10.7)	10 (15.4)	11 (6.6)	1 (0.9)		
Tumor size	<3 cm	87 (62.1)	33 (50.8)	96 (57.8)	67 (63.8)	0.6724	0.6725
	≥3	23 (16.4)	16 (24.6)	32 (19.3)	16 (15.2)		
	Unknown	30 (21.5)	16 (24.6)	38 (22.9)	22 (21.0)		
BCG treatment	No	111 (79.3)	53 (81.5)	134 (80.7)	90 (85.7)	0.6238	0.6725
	Yes	29 (20.7)	12 (18.5)	32 (19.3)	15 (14.3)		
CIS in disease course	No	112 (80)	55 (84.6)	145 (87.3)	90 (85.7)	0.3471	0.6249
	Yes	28 (20)	10 (15.4)	21 (12.7)	15 (14.3)		
Cystectomy	No	135 (96.4)	60 (92.3)	156 (94.0)	93 (88.6)	0.1045	0.3134
	Yes	5 (3.6)	5 (7.7)	10 (6.0)	12 (11.4)		

*BCG, bacillus Calmette-Guérin; CIS, carcinoma in situ; P-values were obtained by Fisher's exact test; FDR was corrected by the Benjamini & Hochberg method*.

### Prognostic Significance of the Four Immune Subtypes

**Figure 5:**
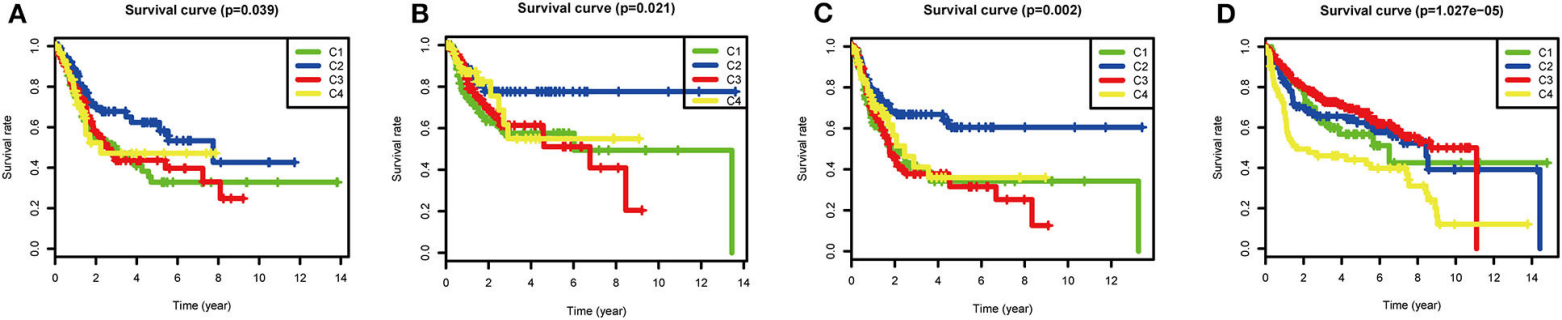
Survival analysis of the four subtypes. Kaplan–Meier survival curves for **(A)** overall survival (OS), **(B)** relapse-free survival (RFS), and **(C)** progression-free survival (PFS) in the TCGA cohort, and **(D)** overall survival (OS) in the GEO cohort. *P* < 0.05 was considered significant.

Although most BLCAs are “superficial,” they recur in 50–75% of cases. The prevalence of BLCA far exceeds its incidence (4, 19). In view of the high rates of recurrence and progression of BLCA, the four subtypes based on ssGSEA scores of immune gene sets were used to investigate the clinical prognosis. In the TCGA cohort, the C2 subtype had the best OS (*P* = 0.039), RFS (*P* = 0.021), and PFS (*P* = 0.002), indicating that high immune infiltration is beneficial for survival in BLCA. In the GEO validation cohort (*P* < 0.001), C2, and C3 had the best OS, and C4 had the worst OS. The high OS for C3 may be explained by a specific GEO dataset with a high frequency of this subtype (Figure 5).

### Comparison of 22 Immune Cells Among the Four Subtypes Using CIBERSORT

**Figure 6:**
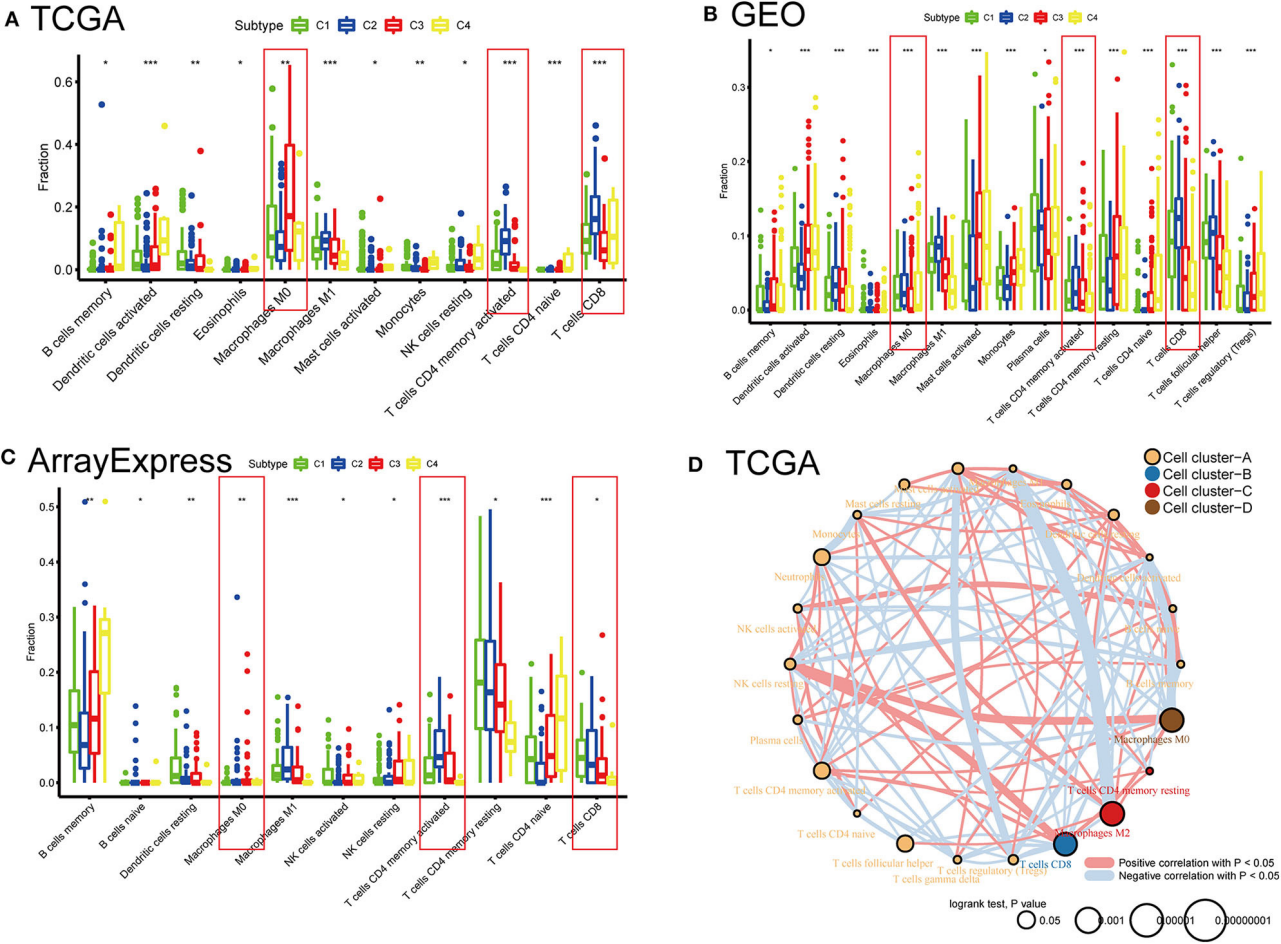
Distribution differences and prognostic significance of the four subtypes in 22 human immune cells using the CIBERSORT algorithm. **(A)** TCGA, **(B)** GEO, and **(C)** ArrayExpress cohorts showed significantly different subgroups of immune cells among the four subtypes. *P < 0.05, **P < 0.01, ***P < 0.001. **(D)** Network diagrams of correlation and prognosis for 22 immune cell subgroups in the TCGA cohort.

To compare the differential distribution of the four subtypes in human immune cell subgroups, we used the CIBERSORT algorithm to calculate the contents of 22 immune cell populations in three datasets for each subtype by setting *p* < 0.05 as the threshold for screening. We also performed correlation and survival analyses of the 22 immune cell types in the TCGA cohort (Figure 6). CD8 T cells, CD4 memory-activated T cells, follicular helper T cells, M0 macrophages, M2 macrophages, and neutrophils had prognostic significance in BLCA. Frequencies of M0 macrophages, CD4 memory-activated T cells, and CD8 T cells differed significantly among the immune subtypes in the three datasets. CD8 T cells and CD4 memory T cells were most frequent in C2 and least frequent in C4 (i.e., C2 > C1 > C3 > C4). This is consistent with our previous results regarding the frequency of CD8 T cells and levels of characteristic genes related to the cytolytic activity (*CD8A* and *GZMB*) in each subtype (Supplementary Figure 2). The subtype distribution of M0 macrophages varied substantially among datasets. In the ArrayExpress and TCGA cohorts, the ratio of M0 macrophages decreased in the order of C3 > C4 > C1 > C2, and the corresponding rank in the GEO cohort was C2 > C1 > C4 > C3. This difference may be explained by the loss of some genes associated with macrophages during the process of data collection.

### Differences in Sensitivity of Immune Subtypes to Immuno/Chemotherapy

**Figure 7:**
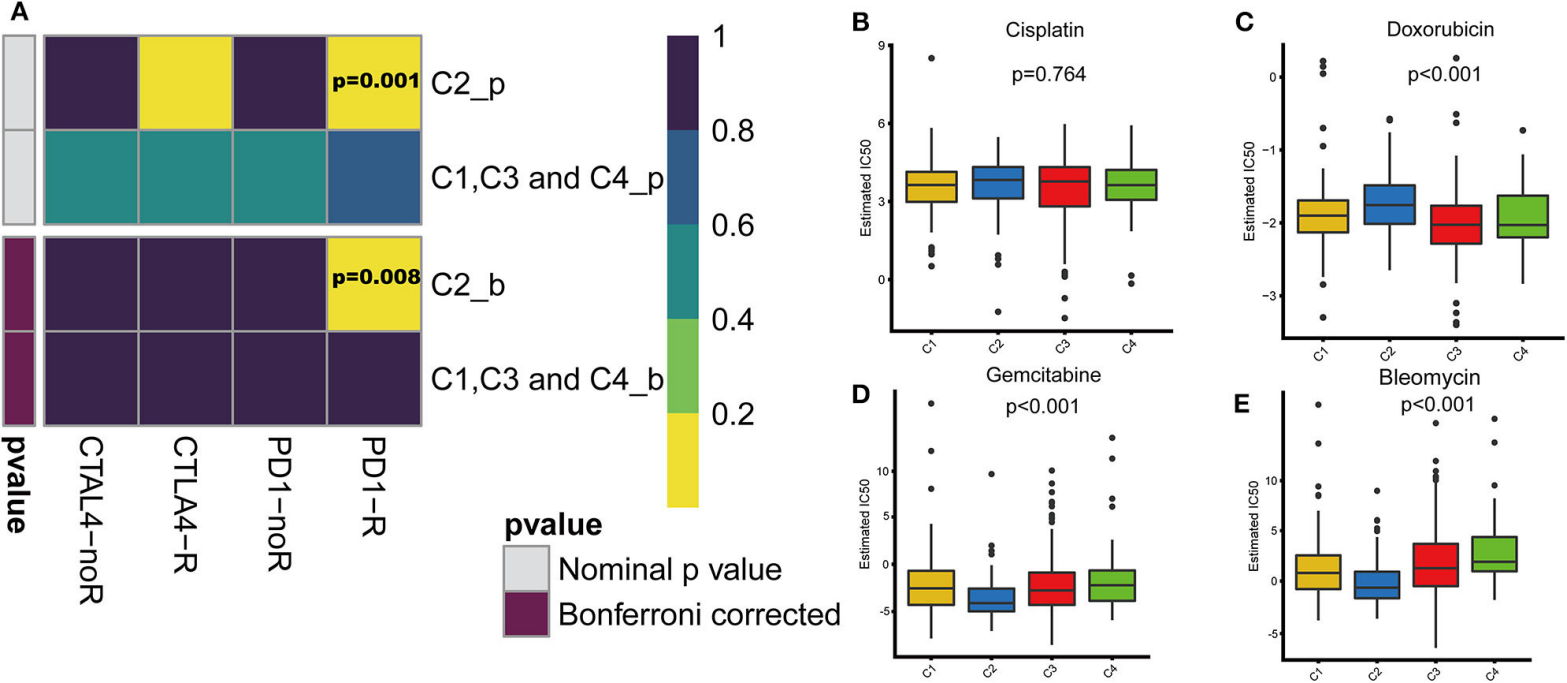
Differences in sensitivity of immune subtypes to immuno/chemotherapy. **(A)** Submap analysis manifested that C2 could be more sensitive to the programmed cell death protein 1 inhibitor (Bonferroni-corrected *P* = 0.008). The box plots of the estimated IC50 for **(B)** cisplatin, **(C)** doxorubicin, **(D)** gemcitabine and **(E)** bleomycin are shown for C1–C4 in TCGA cohort.

With the approval of immune checkpoint inhibitors as routine drugs for BLCA, the possibility of immunotherapy can be further investigated. We used subclass mapping to compare the expression profiles of the four defined immune subtypes with those in a published data set containing 47 melanoma patients who responded to immunotherapy (20, 21). The C2 subtype was more sensitive to anti-PD-1 treatment than other subtypes (Bonferroni correction *P* = 0.008). However, with regard to the conventional chemotherapy of BLCA, the C2 subtype exhibited a response different from that to immunotherapy. We selected four representative chemical drugs (cisplatin, bleomycin, doxorubicin, and gemcitabine) to evaluate the response of the four immune subtypes. We trained the prediction model on the GDSC cell line dataset using ridge regression and evaluated the satisfactory prediction accuracy using 10-fold cross-validation. We estimated the IC50 of each sample in TCGA dataset based on the prediction models of these four chemical drugs. For cisplatin and doxorubicin, C2 was the least sensitive and C4 was most sensitive compared with the other subtypes. For bleomycin and gemcitabine, C2 was most sensitive and C4 was the least sensitive relative to the other subtypes (Figure 7).

### Gene Set Enrichment Analysis

**Figure 8:**
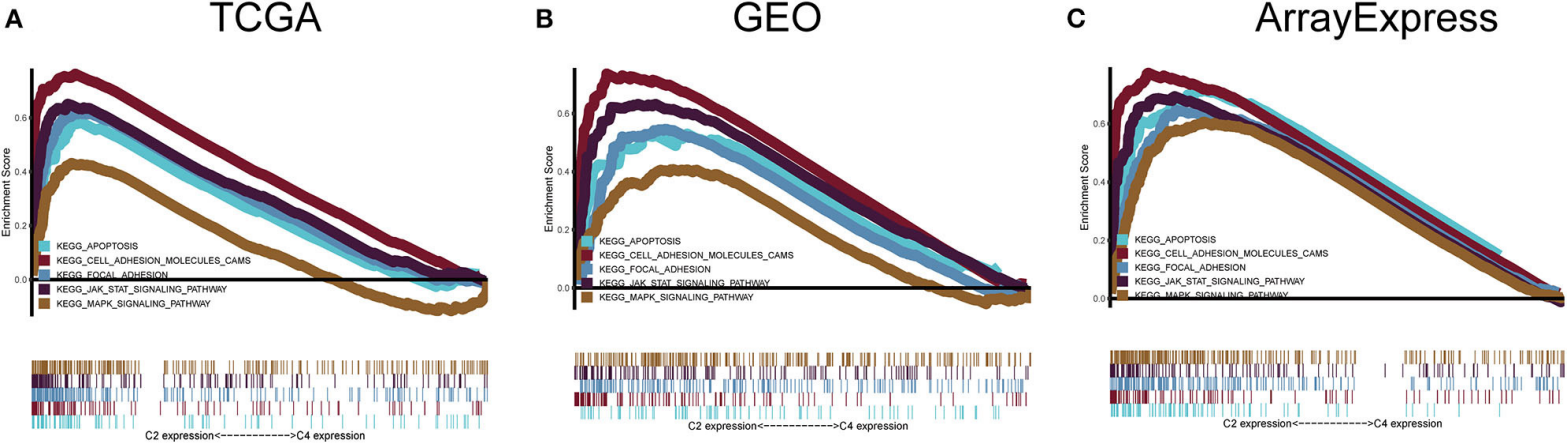
Gene set enrichment analysis. **(A–C)** C2 vs. C4 gene set enrichment analysis (GSEA) in TCGA, GEO, and ArrayExpress cohorts. FDR < 0.05 was the screening threshold.

To explore the biological changes associated with each subtype, we selected the C2 and C4 subtypes for GSEA. The C2 subtype was enriched for genes related to apoptosis, JAK-STAT signaling pathway, the mitogen-activated protein kinase (MAPK) signaling pathway, focal adhesion, and cell adhesion molecules, and these results were verified using GEO and ArrayExpress datasets. C4 was enriched for genes involved in metabolic pathways, but this enrichment was not significant under the strict FDR < 0.05 threshold (Figure 8).

### Somatic Mutation Landscape of Immune Subtype-Related Pathways Identified by GSEA

**Figure 9:**
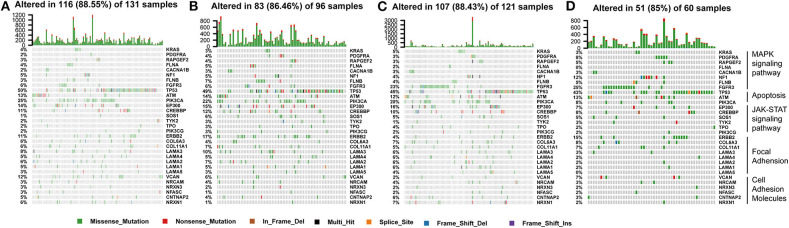
Somatic mutation landscape of immune subtype-related pathways identified by GSEA among **(A–D)** C1-C4 in TCGA cohort.

The tumor genome pattern is reportedly related to antitumor immunity. To investigate whether there is a difference in the frequency of somatic mutations associated with the BLCA subtype-related pathway, and to observe the different patterns of mutations in the BLCA subtypes, somatic mutation data from TCGA database were analyzed. Figure 9 shows genes with high mutation frequency in the MAPK signaling pathway, apoptosis pathway, JAK-STAT signaling pathway, and in focal adhesion and cell adhesion molecules. The frequency of *FGFR3* mutations (23 and 25%) in C3 and C4 was much higher than that in C1 (6%) and C2 (6%). The mutation frequency of *PIK3CA*, an important apoptosis-related gene, in C4 (8%) was significantly lower than that in C1 (25%), C2 (22%), and C3 (20%). The mutation frequency of *EP300*, one of the core genes of the JAK-STAT signaling pathway, in C4 (7%) was significantly lower than that in C1 (14%), C2 (15%), and C3 (16%). In addition, the mutation frequency of *ERBB2* in C3 (4%) was significantly lower than that in C1 (11%), C2 (17%), and C4 (15%).

### Correlation of BLCA Subtypes With Stroma and Stem Cell Characteristics

**Figure 10:**
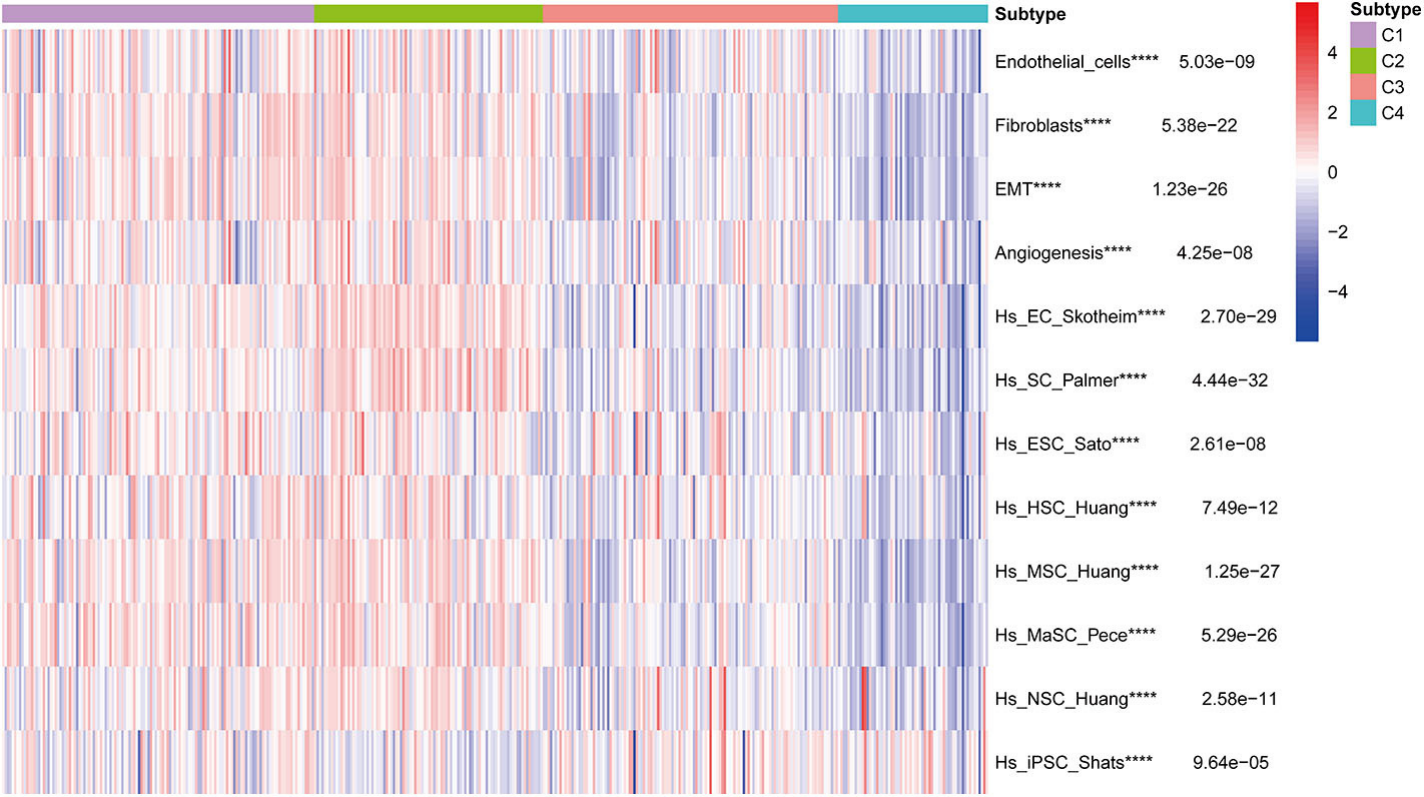
Correlation of BLCA subtypes with stroma and stem cell characteristics. Kruskal-Wallis test, ****P < 0.0001 in TCGA cohort.

Stromal and stem cell infiltration were studied as there are significant differences in stromal scores, in addition to immune scores, of immune subtypes of BLCA. The ssGSEA algorithm was used to calculate the abundance of four stromal components and eight stem cell types, and is illustrated in the heat map (Figure 10). Significant differences were observed between C2 and three other subcategories, including four stromal components (endothelial cells, fibroblasts, EMT, and angiogenesis) and seven stem cell populations [EC (embrional carcinoma), SC (pluripotent stem cell), ESC (Embryonic stem cell), HSC (hematopoietic stem cell), MSC (mesenchymal stem cell), MaSC (mammary epithelial cell), and NSC (neural stem cell)], Which in C2 subtype was significantly higher than the C1, C3, and C4 subtypes. It is worth noting that the stromal components in C2 (endothelial cells and fibroblasts, EMT and angiogenesis) were significantly higher, which is consistent with the previous results of C2-enriched stroma-related features. Furthermore, the abundance of iPSC (induced pluripotent stem cell) in C3 and C4 was significantly higher than that in C1 and C2 (Figure 10).

### Analysis of Gene Co-expression Networks in the Four Subtypes

**Figure 11:**
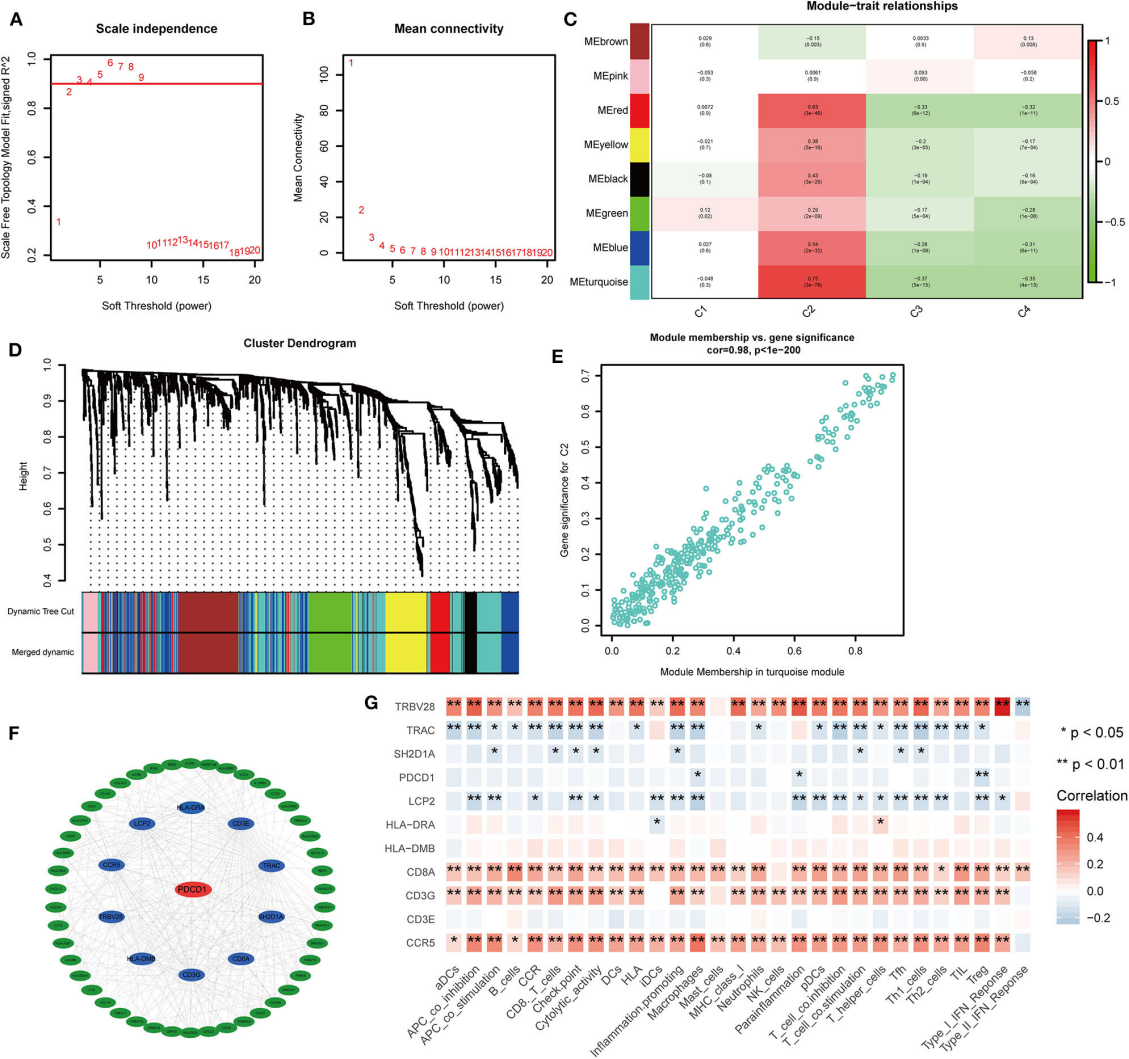
Co-expression analysis of 1,671 immune genes in the TCGA cohort. **(A,B)** Topological network analysis of the optimal soft threshold. **(C)** Dynamic tree cut after module combination. Co-expressed genes can be divided into brown, pink, red, yellow, black, green, blue, and turquoise modules. **(D)** Heatmap of the correlations between the eight modules and four subtypes is shown. **(E)** Heatmap of genes with the most significant correlations with the C2 subtype in the turquoise module. **(F)** Gene interaction network for the turquoise module. **(G)** Correlation heatmap of hub genes and 29 immune gene sets. *P < 0.05, **P < 0.01.

**Figure 12:**
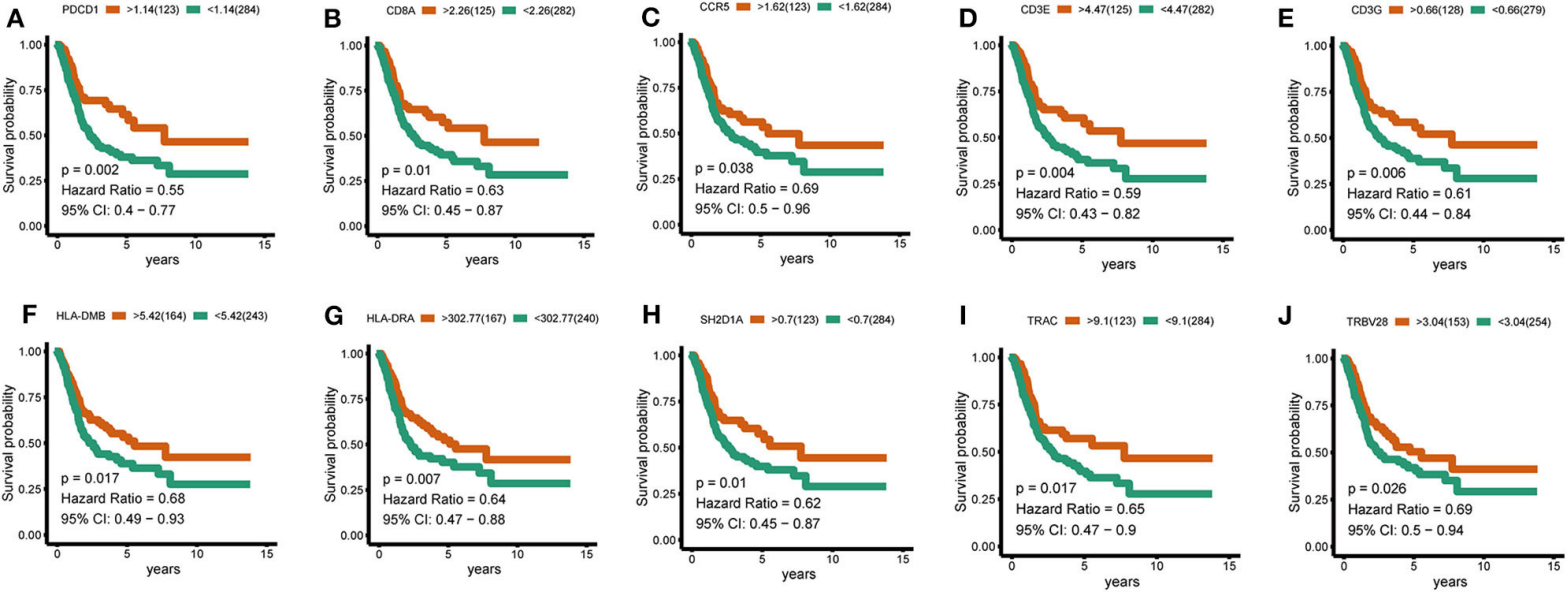
Hub-gene survival analysis. **(A–J)** Survival analysis of 10 hub genes divided according to the optimal thresholds of their respective expression levels. *P* < 0.05 was considered significant.

To further identify the characteristic marker genes for BLCA immunotyping, we downloaded 1,671 immune genes from the IMMPORT database for gene co-expression network analysis (WGCNA) and obtained eight gene modules. Two modules shown in yellow and black in Figure 11 were positively correlated with C2 and negatively correlated with C1, C3, and C4. The red, green, and blue modules positively correlated with C1 and C2, and negatively correlated with C3 and C4. The brown module negatively correlated with C2 and positively correlated with C1, C3, and C4. The pink module negatively correlated with C1 and C4 and positively correlated with C2 and C3. The turquoise module was more highly correlated with C2, C3, and C4 than with C1, with a positive correlation with C2 and inverse correlations with C3, C4, and C1. In the turquoise module, 11 hub genes centered on *PDCD1* were identified, and a strong correlation with the ssGSEA scores for 29 immune gene sets was confirmed. *SH2D1A, PDCD1, LCP2*, and *TRAC* negatively correlated with the majority of immune gene sets, and *TRVB28, HLA-DRA, HLA-DMB, CD8A, CD3E, CD3G*, and *CCR5* positively correlated with the majority of immune gene sets (Figure 11). A survival analysis showed that high levels of all genes except *LCP2* are predictive of a good prognosis for BLCA (Figure 12).

## Discussion

BLCA is a highly aggressive malignant tumor. In the United States, it is the fourth leading cancer type and the seventh leading cause of death in men, and the eighth leading type of cancer and the tenth leading cause of death in women (19). Molecular subtypes of BLCA, generally based on gene expression profiles, have been or will soon be incorporated into clinical management. We focused on the immunotyping of BLCA based on specific immune gene sets and validated the reliability and reproducibility of our findings.

Among the four distinct subtypes identified in our study, C2 was an immune-infiltrating type, and C4 was an immune “desert” type. The C2 and C4 subtypes were associated with upregulation and downregulation, respectively, of numerous genes encoding immune checkpoints and HLA molecules, as well as other immune cell signatures. Furthermore, survival analysis showed that C2 had a better prognosis than the other subtypes. In the GSEA, in addition to enrichment for immune-related pathways, the C2 subtype was enriched for many pathways closely related to the occurrence, development, and metastasis of cancer, such as apoptosis, cell adhesion, and focal adhesion, as well as JAK-STAT and MAPK signaling pathways (22–27). Activation point mutations of *FGFR3* are found in up to 80% of low-grade and staged urothelial carcinomas (UC) of the bladder (28). The most common mutations tend to involve ligand-independent receptor dimerization, which lead to transphosphorylation and downstream signaling. In telomerase-immortalized human urothelial cells (TERT-NHUC) expressing the *FGFR3–TACC3* fusion gene, a point mutation in *FGFR3* (S249C) activates the MAPK pathway and phospholipase cγ1 (PLCγ1), and induces PLCγ1-dependent overgrowth at the confluence (28, 29). At the same time, other studies have shown that a *FGFR3* mutation suppressed acute inflammation, causing immune cells (mainly neutrophils) to behave aberrantly in tumors, thereby causing tumor progression (30). The high-frequency mutations of FGFR3 in the C3 and C4 subtypes are likely to be the cause of poor prognosis. *PIK3CA* encodes the p110α catalytic subunit of phosphatidylinositol 3-kinase (PI3K). It regulates important cellular functions via the PI3K/Akt pathway, including proliferation, metabolism, protein synthesis, angiogenesis, and apoptosis. It is known that mutations in *PIK3CA* are related to a variety of human cancers, and the mutant *PIK3CA* is considered an oncogene. Evidence shows that the pan-PI3K inhibitor BKM120 significantly inhibits the growth of human BLCA cell lines with *PIK3CA* mutations, and the addition of BKM120 makes *PIK3CA*-mutated tumors sensitive to PD-1 blockage (31, 32). This is consistent with the higher rate of *PIK3CA* mutations in the C2 subtype and its increased sensitivity to anti-PD-1 therapy. EP300 and CREB binding protein (CREBBP) are two homologous lysine acetyltransferases (KAT) in metamorphoses, which have multiple cellular functions. They mainly function as transcriptional regulators, but also exert non-transcriptional effects on DNA replication and metabolism, inside and outside the nucleus. Although *EP300*/*CREBBP*-inactivating mutations (33, 34) have been observed in some cancers, secondary gain-of-function mutations in *EP300*/*CREBBP* may further propel cancer development (35). Nevertheless, it is not clear how the increase in the KAT activity of EP300 or CREBBP promotes malignant tumors. *ERBB2* is reportedly extensively mutated in solid tumors. Preclinical data indicate that *ERBB2*-activating mutations are responsive to ERBB2 tyrosine kinase inhibitors (36). The latest results indicate that the clinical benefit of ERBB2 tyrosine kinase inhibitor, neratinib, may depend on the type of *ERBB2* mutation and the type of tumor. For example, no response to neratinib treatment was observed in BLCA and colorectal cancer patients with *ERBB2* mutations in a clinical study (37). Therefore, the significance and mechanism of *EP300, CREBBP*, and *ERBB2* mutations in BLCA warrant in depth-investigation.

C2 was associated with the most potent immune infiltration and antitumor properties, including high neutrophil, B cell, CD8+ T cell, and macrophage infiltration. These results were verified using the CIBERSORT algorithm, evaluating 22 human immune cell subgroups. For the three platforms (TCGA, GEO, and ArrayExpress), the distributions of immune cell subgroups, and survival related to M0 macrophages, CD4 memory-activated T cells, and CD8 T cells significantly differed among the four subtypes. The proportion of CD4 memory-activated T cells and CD8 T cells was much higher in C2 than in the other subtypes, and the proportion of M0 macrophages was lower in C2 than in the other subtypes. The antitumor effects of CD4 memory-activated T cells and CD8 T cells in the immune system are established. However, in addition to the immune functions of tumor-associated macrophages (TAMs), extensive research has shown that TAMs regulate tumor growth, invasion, metastasis, extracellular matrix remodeling, and angiogenesis via the release of epidermal growth factor, chemokines, *MMP*, and *VEGF*. The density of TAMs is associated with a poor prognosis (38–41). This is consistent with the observed downregulation and upregulation of M0 macrophages in C2 and C4 subtypes in the TCGA cohort, and is also consistent with the prolonged survival of the C2 subtype compared to other subtypes.

In our study, we demonstrated that a stronger immune infiltration subtype C2 could indeed predict better OS and lower relapse rates. Moreover, C2 showed the strongest sensitivity to anti-PD-1 treatment compared to other subtypes. C2 subtype benefits from PD-1 inhibitor treatment as it hinders the interaction between PD-1 and PD-L1, thereby enhancing *in vitro* T cell response and mediating preclinical anti-tumor activity. Moreover, Aptsiauri et al. (42) pointed out that the transition of primary tumors from HLA-1-positive to HLA-1-negative (MHC/HLA class I loss in cancer) is one of the main mechanisms for tumors to escape from T cell recognition and destruction (43). This is in line with our observations that HLA molecules are generally upregulated in C2 subtype and downregulated in C4 subtype of BLCA. The different immune cell infiltration states of the subtypes are also in agreement with the observed tumor heterogeneity in the early (permissible phase) stage of BLCA where HLAI-positive and HLA-negative tumor cells are present, wherein tumor infiltrating lymphocytes and M1 macrophages respond as part of the active anti-tumor Th1. In the later stage (encapsulation period), tumor nests are mostly HLA-I-negative with immune cells residing in the peri-tumoral stroma, which forms a granuloma-like encapsulated tissue structure. In addition, the loss of heterozygosity (LOH) in the chromosome region 6p21.3 may lead to the loss of HLA haplotypes, which is considered to be an important mechanism for tumors to escape from T lymphocyte recognition (44), and may be the reason for the poor effect of immunotherapy in C4 subtype. The C2 subtype was most sensitive to treatment with conventional chemotherapeutic drugs such as gemcitabine and bleomycin, whereas it was least sensitive to cisplatin and doxorubicin. In contrast, the C4 subtype was most sensitive to cisplatin and doxorubicin. The above discussion reveals that the patients of C2 subtype of BLCA may benefit from a combination of chemotherapy and immunotherapy. This study aims to provide substantiation to the need for personalized and precise treatment in clinical practice.

Finally, in a WGCNA, hub genes related to subtype characteristics were identified. Eleven hub genes centered on PDCD1 positively correlated with the C2 subtype, negatively correlated with the C3 and C4 subtypes, and were closely related to comprehensive immune responses. A survival analysis revealed that high levels of *SH2D1A, PDCD1, TRAC, TRVB28, HLA-DRA, HLA-DMB, CD8A, CD3E, CD3G*, and *CCR5* were predictive of a good prognosis for BLCA, consistent with the better survival probability for the C2 subtype compared to subtypes. The discovery of these immune-related genes may be good news for patients with immune “desert” phenotypes. If the expression of these immune genes in the “desert” -like phenotype is upregulated, it may significantly enhance the outcome of immunotherapy. These results highlight the vital role of these genes in the immune environment.

Our study has some limitations. For instance, in the GSEA, non-immune pathways significantly related to the C4 subtype were not enriched, which limited our analysis of the characteristics of the C4 subtype associated with tumor pathways. The results also showed bias toward the C2 subtype. Additionally, based on the 29 immune gene sets, immunophenotyping did not simultaneously reflect all immune characteristics of BLCA. Some potentially important immune cells were not included, for example, no gene sets were defined for central memory T cells, which produce antibodies with long-term memory after antigen activation. Moreover, the interaction of immune gene sets was not included in this study, and this should be a focus of future research.

In conclusion, the immunotyping of BLCA based on immune gene sets clearly described the heterogeneity of different BLCA immune microenvironments, which reflected the sensitivity of immunotyping. This study also revealed that the occurrence and development of BLCA were considerably affected by the immune microenvironment. Validation of different immune-related methods may provide clinical decisions for BLCA, as well as other cancers.

## Data Availability Statement

The datasets presented in this study can be found in online repositories. The names of the repository/repositories and accession number(s) can be found in the article/Supplementary Material.

## Author Contributions

CT and JM: design, analysis, interpretation of data, drafting of the manuscript, and critical revision of the manuscript. XL: statistical analysis. XL and ZL: critical revision of the manuscript for important intellectual content, administrative support, obtaining funding, and supervision. All authors read and approved the final manuscript.

## Conflict of Interest

The authors declare that the research was conducted in the absence of any commercial or financial relationships that could be construed as a potential conflict of interest.

## Supplementary Material

Supplementary Figure 1 Heatmap of the TCGA cohort of bladder cancer subtypes.

Supplementary Figure 2 Differences in HLA gene expression among bladder cancer immune subtypes. Distribution of HLA gene expression in the four subtypes in the TCGA and **(B)** ArrayExpress cohorts. Differences in molecular subtypes of BLCA in each dataset were compared using the Kruskal–Wallis test. ^***^*P* < 0.001.

Supplementary Figure 3 Differences in CD8 T cell and macrophage marker gene expression among the four subtypes. **(A–C)** Differences in CD8A expression among the four subtypes in the TCGA, GEO, and ArrayExpress cohorts. **(D–F)** Differences in GZMB expression among the four subtypes in the TCGA, GEO, and ArrayExpress cohorts; bars indicate medians. ^***^*P* < 0.001.
