# Supplementary figures and images for: Identification of Four Immune Subtypes in Bladder Cancer Based on Immune Gene Sets
Source: Front Oncol. 2020 Oct 5;10:544610. doi: 10.3389/fonc.2020.544610 (PMC7571508; doi:10.3389/fonc.2020.544610)

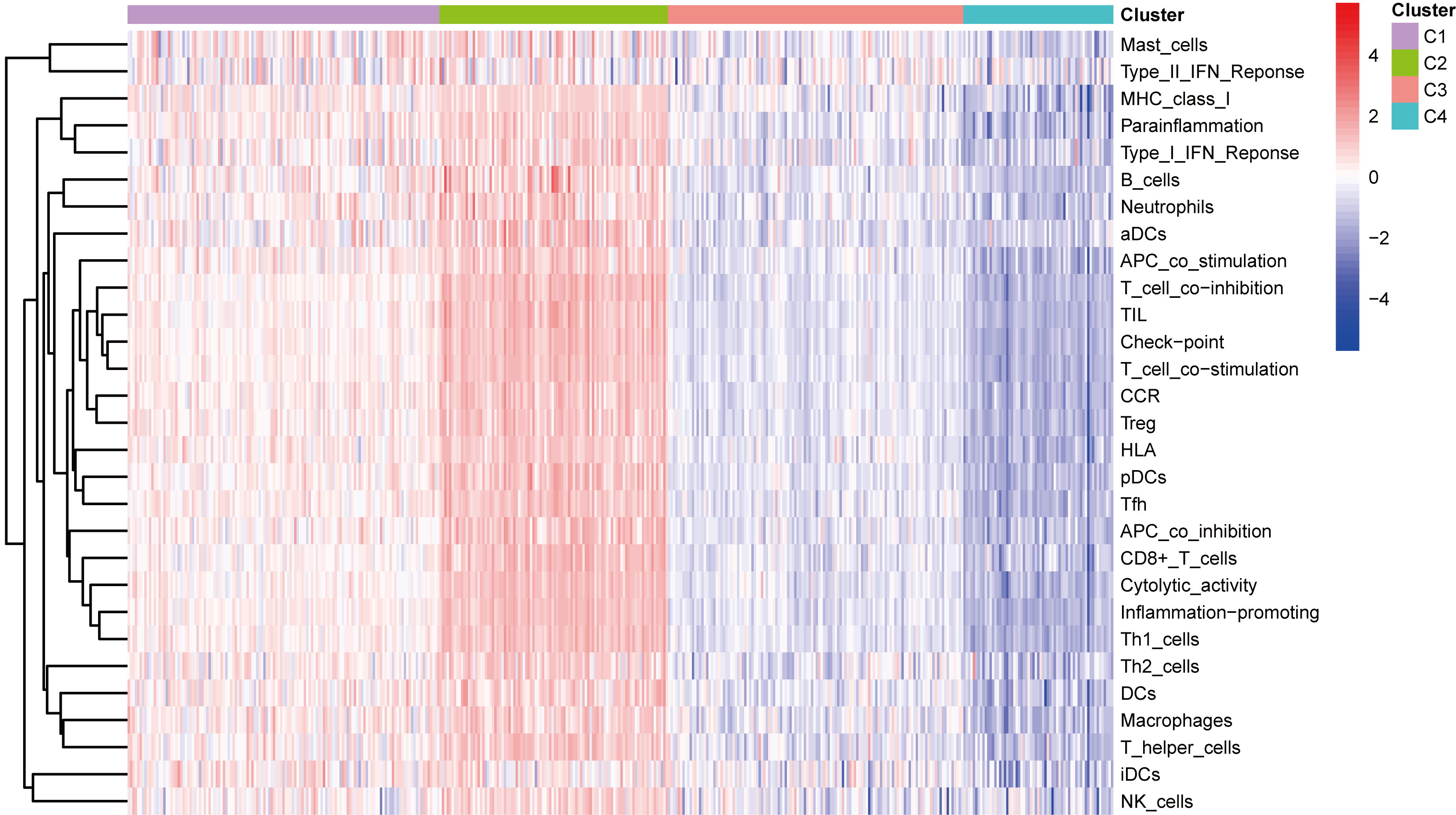

Supplement: Supplementary Figure 1 — Heatmap of the TCGA cohort of bladder cancer subtypes. [file Image_1.TIF]

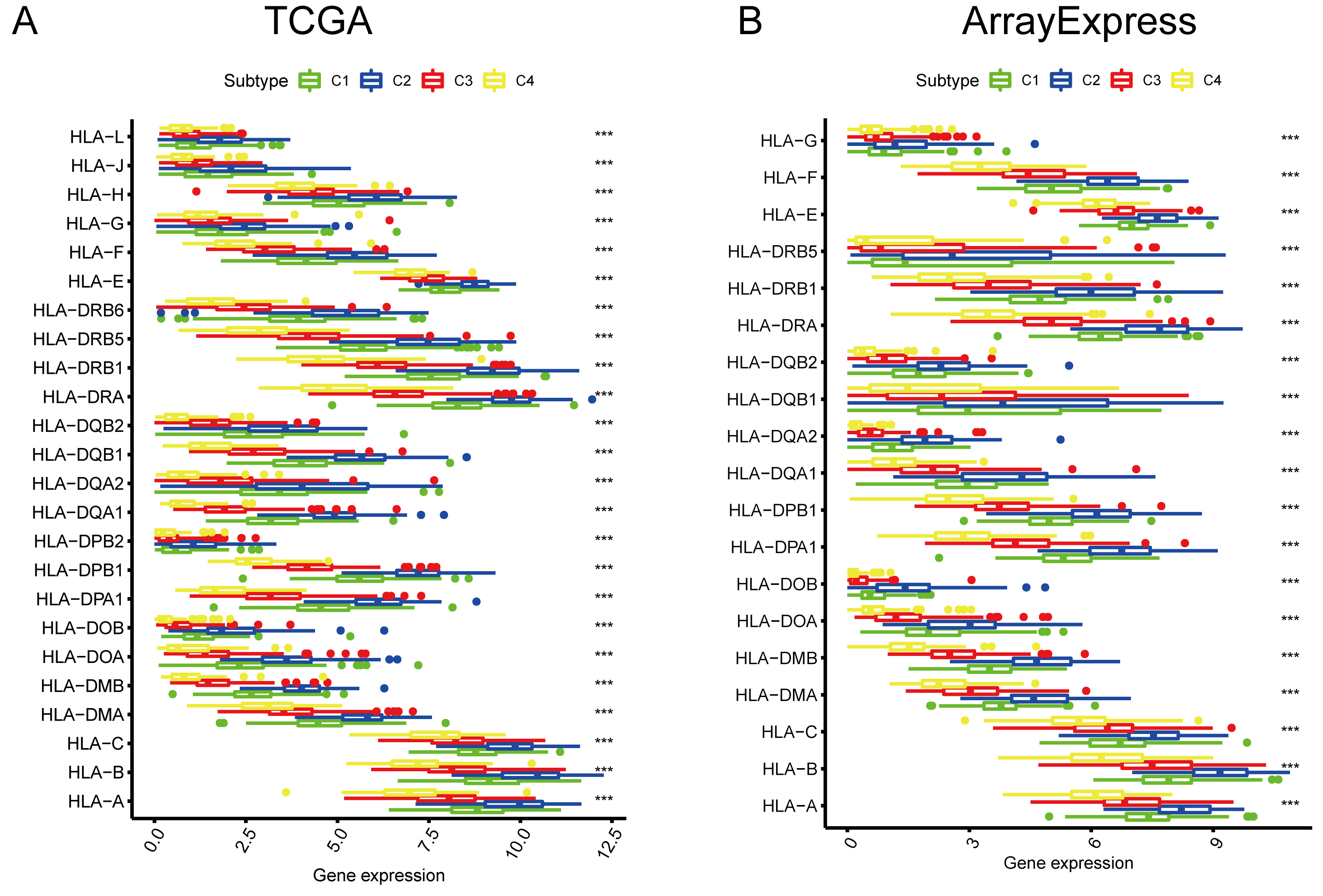

Supplement: Supplementary Figure 2 — Differences in HLA gene expression among bladder cancer immune subtypes. Distribution of HLA gene expression in the four subtypes in the TCGA and (B) ArrayExpress cohorts. Differences in molecular subtypes of BLCA in each dataset were compared using the Kruskal–Wallis test. ***P < 0.001. [file Image_2.TIF]

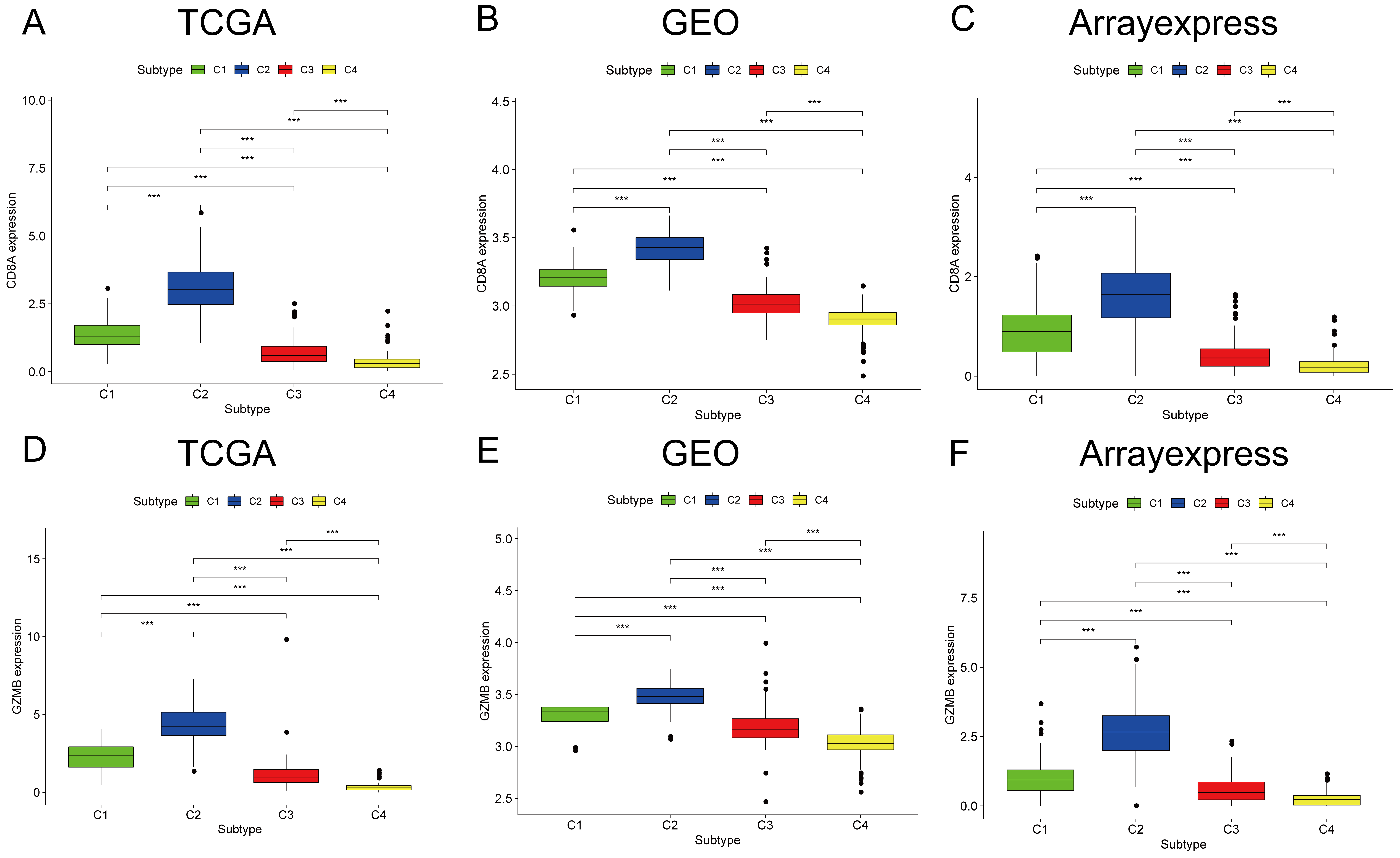

Supplement: Supplementary Figure 3 — Differences in CD8 T cell and macrophage marker gene expression among the four subtypes. (A–C) Differences in CD8A expression among the four subtypes in the TCGA, GEO, and ArrayExpress cohorts. (D–F) Differences in GZMB expression among the four subtypes in the TCGA, GEO, and ArrayExpress cohorts; bars indicate medians. ***P < 0.001. [file Image_3.TIF]
